# Mathematical Modelling of Immune Parameters in the Evolution of Severe Dengue

**DOI:** 10.1155/2017/2187390

**Published:** 2017-02-15

**Authors:** M. K. Premaratne, S. S. N. Perera, G. N. Malavige, Saroj Jayasinghe

**Affiliations:** ^1^Research and Development Center for Mathematical Modeling, Faculty of Science, University of Colombo, Colombo, Sri Lanka; ^2^Centre for Dengue Research, Faculty of Medicine, University of Sri Jayewardenepura, Nugegoda, Sri Lanka; ^3^Department of Clinical Medicine, Faculty of Medicine, University of Colombo, Colombo, Sri Lanka

## Abstract

*Aims*. Predicting the risk of severity at an early stage in an individual patient will be invaluable in preventing morbidity and mortality caused by dengue. We hypothesized that such predictions are possible by analyzing multiple parameters using mathematical modeling.* Methodology*. Data from 11 adult patients with dengue fever (DF) and 25 patients with dengue hemorrhagic fever (DHF) were analyzed. Multivariate statistical analysis was performed to study the characteristics and interactions of parameters using dengue NS1 antigen levels, dengue IgG antibody levels, platelet counts, and lymphocyte counts. Fuzzy logic fundamentals were used to map the risk of developing severe forms of dengue. The cumulative effects of the parameters were incorporated using the Hamacher and the OWA operators.* Results*. The operator classified the patients according to the severity level during the time period of 96 hours to 120 hours after the onset of fever. The accuracy ranged from 53% to 89%.* Conclusion*. The results show a robust mathematical model that explains the evolution from dengue to its serious forms in individual patients. The model allows prediction of severe cases of dengue which could be useful for optimal management of patients during a dengue outbreak. Further analysis of the model may also deepen our understanding of the pathways towards severe illness.

## 1. Introduction

Sri Lanka has experienced a marked increase in dengue from 2004 to 2014. The increase from the year 2013 to 2014 was 47.4% (from 32,063 to 47,246) [1]. Although the overall mortality rate is low, every patient carries the potential risks of developing dengue hemorrhagic fever (DHF). The current case fatality is around 0.2–0.3% and early diagnosis and optimum care should be able to reduce these figures further. Under these circumstances it would be greatly beneficial if DHF could be predicted early.

The infection is spread by a mosquito that acts as the vector for the disease. After entering the body from a bite, the incubation period lasts for about 4–10 days. Although the majority of the infected individuals develop asymptomatic disease, approximately 10% develop symptoms such as high fever, diarrhea, retroorbital pain, joint and muscle pains, or more severe forms of clinical disease associated with fluid leakage [2].

With the symptomatic febrile phase there are changes in the platelet count, lymphocyte count, NS1 antigen levels, IgG antibody levels, and other parameters including cytokines levels. After a variable period (about 3 to 5 days) a minority of symptomatic patients enter a phase of fluid loss from the intravascular compartment through capillaries (i.e., called the “critical phase” to denote its importance in management). This stage lasts for 48 hours and it is crucial that patient receive adequate amounts of fluid to counter the fluid leak during this period. If the degree of severity increases, the organs of the patient gradually fail resulting in the death of the patient.

The mediation of fluid leak is through cytokines acting on the vascular endothelium, and currently there is evidence to link it to platelet activating factor (PAF) [3].

One marker of severity is the persistence of the dengue NS1 antigen. This indicates the presence of dengue virus and its presence during the period of 5-6 days after illness is associated with more severe disease [4]. Dengue NS1 itself appears to be associated with vascular leak and triggers the production of many inflammatory cytokines.

The virus has the ability to destroy platelets and reduce its production in the marrow thus reducing the platelet count [5]. Hence the platelet count is a significant marker in determining the severity of dengue and a count less than 20,000 platelets/mm^3^ is an indicator of severe dengue [6].

The severity of illness increases depending on whether it is the primary or the secondary infection, and in the latter the antibodies developed due to the primary dengue infection stimulate a stronger immune mediated illness (i.e., known as antibody dependent enhancement) [7]. The changes in the dengue virus specific IgG reveal whether the patient is having a secondary or a primary infection. High levels of dengue virus specific IgG indicate a secondary infection [6]. The lymphocyte indicates how the body is recovering from the disease. A lymphocyte count below 1,500 cells/mm^3^ is associated with more severe stages of dengue [6].

Viral dynamics during primary and secondary infections has been researched upon [8] and these viral dynamic models elaborate on the immune response and its dynamics in the body. However most of the researches focus on the effects of a single parameter on the severity of dengue. This study is an attempt to develop a mathematical model based on the cumulative effects of risks indicated by platelet count, lymphocyte count, dengue NS1 antigen, and dengue virus specific IgG levels and other parameters from published data.

## 2. Methodology

### 2.1. Preliminary Analysis of Data

Data was obtained from a previously published patient database where each clinical parameter was collected for multiple time points with twelve-hour time gaps [9]. Data from 25 DHF patients and 11 DF patients were analyzed representing a repeated measures structure.  Table 1 summarizes the descriptive analysis of the data set [9].

The behaviour of dengue specific IgG antibody shows an increase throughout the disease and it attains a stable level after the convalescent phase which has been shown in other studies [10, 11].  Table 1 shows that the median IgG levels of DHF patients are above 22 which implying that majority are having a secondary infection. This can be expected as the antibody dependent enhancement phenomenon increases the risk of developing severe forms of dengue. The median IgG levels of DF patients also increase and exceed 22 suggesting that there are nonsevere dengue patients with secondary infection. DHF and DF patients display a rise in the lymphocyte count. However in DF patients they continue to rise and after 120 hours the levels are significantly higher than in DHF. Both types of patients show a decrease in platelet counts over time, but there is a significant lower median level in DHF. Median values of the NS1 antigen levels do not fluctuate significantly, and NS1 remains positive after the 5th day from the onset of fever for DHF patients, while DF patients show a considerable decline in the NS1 Panbio levels [9].

#### 2.1.1. Interaction between Variables

In order to analyze the interaction between variables, hierarchical clustering for DHF and DF patients at different time points is carried out. According to the availability of data and due to the fact that we need to construct a predictive model for severity we are using the time points 120 h and 132 h as our markers. The clustering has been performed using all the parameters available in the data set.

**Figure 1:**
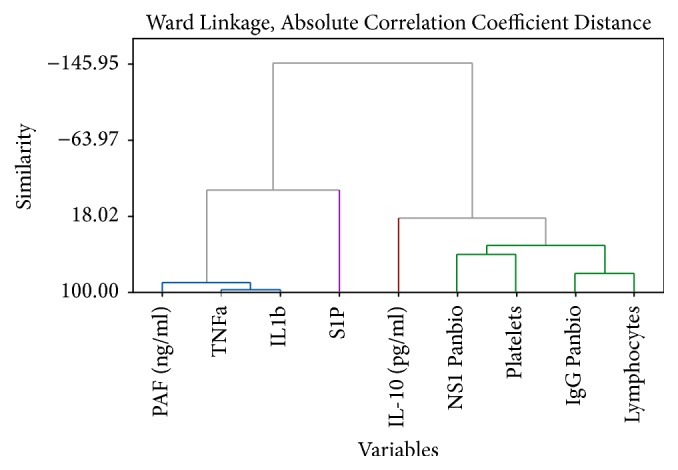
Dendrogram for DHF patients after 120 hours from the onset of fever.

**Figure 2:**
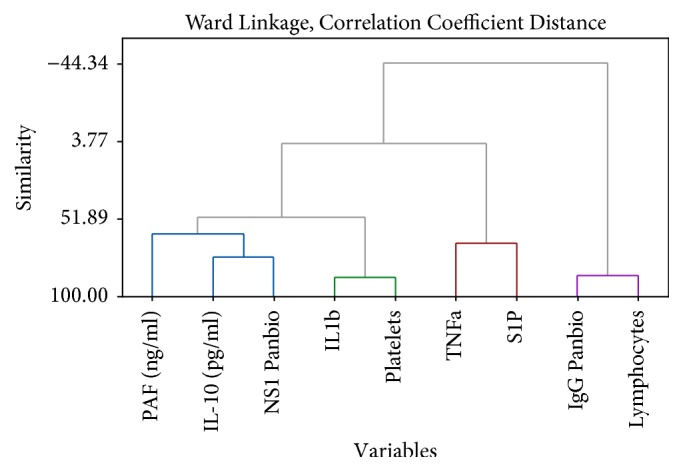
Dendrogram for DHF patients after 132 hours from the onset of fever.

According to Figures 1 and 2, the DHF patients indicate a relationship between the platelet count and the NS1 antigen levels. Furthermore the IgG Panbio units have a similarity with the lymphocyte count. Similar behaviour between the parameters allows us to partition these parameters into two distinct groups.

#### 2.1.2. Ambiguous Parameter Levels

Another important observation to be made is the parameter levels at which the severity of the patient is ambiguous. These parameter levels are used in the mathematical model in order to differentiate between the severe and nonsevere patients (Table 2).

A platelet count less than 50,000 cells/mm^3^ indicates a higher risk of moving into severe forms of dengue [12]. Thus a platelet count of 70,000 cells/mm^3^ [13] which is the median platelet count when the patients are admitted is considered as the lower limit of the ambiguous region. Considering the median platelet count of the patients in the data set for our research the upper limit for the ambiguous region of the platelet count is set at 95,000 cells/mm^3^. The NS1 antigen being positive after 120 hours from the onset of fever indicates increased severity [4]. Therefore, a range of NS1 antigen that indicates an ambiguity of remaining positive after 120 hours is suited for the ambiguous region. For this purpose, we have considered the range in which the central measures of the NS1 data have been distributed in the period before 120 hours. Thus, the range of ambiguity lies in the interval of 25 to 40 NS1 antigen level.

According to the data available on lymphocyte count in the background research [14] the lymphocyte count varies between 2,500 and 4,000 for patients during admission hence the level of severity of the patient is ambiguous in this range. Since the patients with primary infections can develop severe forms we are setting the lower limit at 16 Panbio units. Furthermore, since there are patients with secondary infection who have not developed severe forms of dengue the upper limit of the ambiguous region for IgG is set at 36 Panbio units. The ambiguous region for IgG is also based on the central measures of IgG levels before 120 hours for both DF and DHF patients.

### 2.2. Theoretical Framework

#### 2.2.1. Fuzzy Membership Functions

In a wider sense the fuzzy logic principles are based on the fuzzy set theory [15]. In fuzzy logic a fuzzy subset *F* of a set *A* is defined using a membership function *μ* : *A* → [0,1]. The fuzzy membership function determines the degree to which an element belongs to a defined set.

#### 2.2.2. Fuzzy Operators

A fuzzy operator can be defined as a function *f* : [0,1]^*n*^ → [0,1]. For this study we use the Hamacher operator along with the Ordered Weighted Aggregation (OWA) operator.

Hamacher operator is defined as follows:

(1) Hamacher  product=0if  μAx=μBy=0μAx∗μByμAx+μBy−μAx∗μByotherwise,

where *μ*_*A*_(*x*), *μ*_*B*_(*y*) are membership functions defined on sets *A* and *B*.

The Ordered Weighted Aggregation operator (OWA operator) [16] is defined as follows:

(2) $$\begin{array}{c} OWA\;\;operator=\overset{n}{\underset{i=1}{\sum }}w_{i}*x_{i}, \end{array}$$

where *w*_*i*_ ∈ (*w*_1_, *w*_2_, *w*_3_,…, *w*_*n*_) and *w*_*i*_ is the *i*th element in the weights vector where weight values are arranged in the descending order. *x*_*i*_ denotes the *i*th largest membership value.

The weights vector is defined according to the following conditions and constraints.

(3) $$\begin{array}{c} \overset{n}{\underset{i=1}{\sum }}w_{i}=1. \end{array}$$

The weights of the OWA operator are derived using the linguistic quantifier defined by Xu [17]. The linguistic quantifier is as follows:

(4) $$\begin{array}{c} Q\left(\;r\;\right)=\left\{\begin{array}{cc} 0 & if\;\;r<a\\ \frac{r-a}{b-a} & if\;\;a<r<b\\ 1 & if\;\;r>b, \end{array}\right. \end{array}$$

where *a*, *b*, *r* ∈ [0,1]. Hence the weights of the OWA operator are defined as in

(5) $$\begin{array}{c} w_{i}=Q\left(\;\frac{i}{n}\;\right)-Q\left(\;\frac{i-1}{n}\;\right);\;i=1,2,\ldots ,n. \end{array}$$

Orness of the OWA operator which indicates how close the OWA operator is to an OR operator is defined according to

(6) $$\begin{array}{c} orness=\frac{1}{n-1}\overset{n}{\underset{i=1}{\sum }}\left[\;\left(\;n-i\;\right)*w_{i}\;\right]. \end{array}$$

The principles of concentration and dilution are also used in the model. A membership value is concentrated or diluted according to its relative importance compared to the other parameters in the model. Concentration or the dilution of a membership value is defined as follows:

(7) μxconc=μxγwhere  γ>1μxdiluted=μxγwhere  γ<1.

### 2.3. Mathematical Modelling

#### 2.3.1. Membership Functions

*Membership Function for Platelets*

(8) $$\begin{array}{c} \mu_{P}\left(\;x_{p}\;\right)=\left\{\begin{array}{cc} 0 & if\;\;x_{p}\leq 25\\ 2{\left(\frac{x-25}{125}\right)}^{2} & if\;\;25<x_{p}\leq 87.5\\ 1-2{\left(\frac{x-150}{125}\right)}^{2} & if\;\;87.5<x_{p}\leq 150\\ 1 & if\;\;x_{p}>150. \end{array}\right. \end{array}$$

Severity starts to increase rapidly once the platelet level reduces below 100,000 cells/mm^3^ [12]. This fact is indicated by the rapid increase in the severity after the platelet count drops below 100,000 cells/mm^3^ and the severity level continuously increases as the platelet count drops.

*Membership Function for NS1 Panbio*

(9) $$\begin{array}{c} \mu_{N}\left(\;x_{N}\;\right)=\left\{\begin{array}{cc} 1 & if\;\;x_{N}=0\\ 1-2{\left(\frac{x}{75}\right)}^{2} & if\;\;0<x_{N}\leq 37.5\\ 2{\left(\frac{x-75}{75}\right)}^{2} & if\;\;37.5<x_{N}\leq 75\\ 0 & if\;\;x_{N}>75. \end{array}\right. \end{array}$$

NS1 antigen being positive (i.e., NS1 Panbio greater than 11) after 120 hours after the onset of fever increases the possibility of developing severe forms of dengue. As the NS1 Panbio units increase, the possibility of NS1 remaining positive after 5 days from the onset of fever increases resulting in increased severity hence the membership function for NS1 given above is justifiable [4] (Figure 5).

*Membership Function for Lymphocyte Count*. The background research about the lymphocyte count during a dengue infection indicates that a lymphocyte count less than 1500 indicates a higher severity [6].

Thus the membership function should indicate a higher severity for values less than 1500. Furthermore as the lymphocyte count increases the severity level reduces.

(10) $$\begin{array}{c} \mu_{L}\left(\;x_{L}\;\right)=\left\{\begin{array}{cc} \frac{x}{14000}-\frac{1}{140} & if\;\;100<x_{L}\leq 1500\\ 0.9*\frac{x}{4500}-0.2 & if\;\;1500<x_{L}\leq 6000\\ 1 & if\;\;x_{L}>6000. \end{array}\right. \end{array}$$

*Membership Function for IgG Panbio*

(11) $$\begin{array}{c} \mu_{I}\left(\;x_{I}\;\right)=\left\{\begin{array}{cc} \frac{-x}{36}+1 & if\;\;x_{I}<18\\ 0.5 & if\;\;18<x_{I}\leq 22\\ \frac{-x}{156}+\frac{25}{39} & if\;\;22<x_{I}\leq 100\\ 0 & if\;\;x_{I}>100. \end{array}\right. \end{array}$$

**Figure 3:**
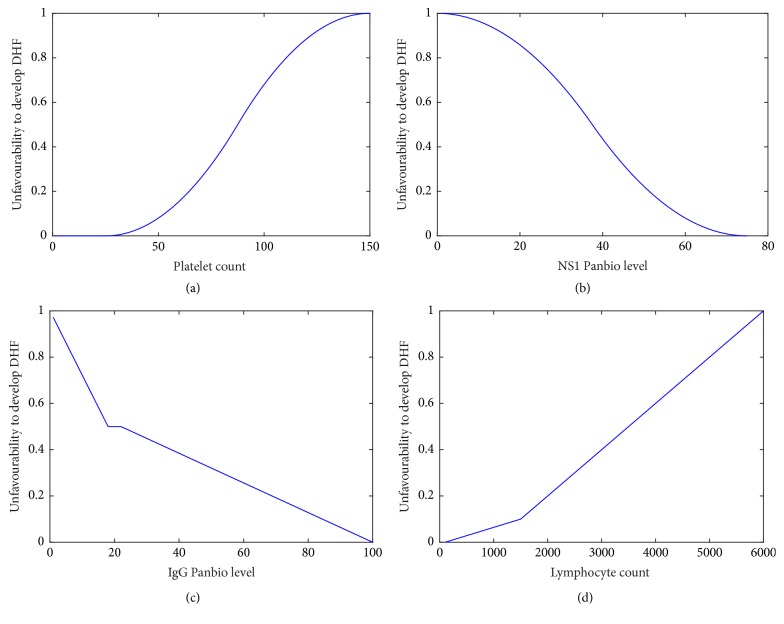
Membership function of the platelet count (a). Membership function of the NS1 Panbio units (b). Membership function of the IgG Panbio units (c). Membership function of the lymphocyte count (d).

This membership function is based on the fact that as the IgG Panbio level increases the risk of developing severe forms of dengue increases due to the secondary infection indicated by IgG Panbio units exceeding 22 (according to the Panbio kit used for this research).  Figure 3 illustrates the four membership functions.

#### 2.3.2. Cumulative Effect

Operator construction depends on the characteristics of the parameters observed through the statistical analysis and the background research.

Our aim is to capture the combined effect of the parameters. Hence the operator should satisfy several conditions. Let *A* and *B* be two clinical parameters and let *U*_*A*_(*x*) and *U*_*B*_(*x*) denote the corresponding degree of favorability to develop DHF.

(1) If *A* and *B* are favorable to develop DHF, then
  (12) if  UAx1,  UBx<1  then  UA∩Bx<min⁡UAx,UBx.
(2) Suppose *U*_*A*_(*x*) < *U*_*B*_(*x*) < 1. Then the effect of an increase in the favorability of *A* (i.e., a decrease in *U*_*A*_(*x*)) on *U*_*A*∩*B*_(*x*) may depend on *U*_*B*_(*x*).
(3) Suppose *U*_*A*_(*x*) and *U*_*B*_(*x*) < 1. The effect of a decrease of *U*_*A*_(*x*) on *U*_*A*∩*B*_(*x*) can be negated by an increase in *U*_*B*_(*x*), that is, an increase in the unfavorability to develop DHF through *B*.

Accordingly we have chosen the Hamacher operator to be applied to each of the clusters of parameters as it proves to be a suitable function [18, 19].

The modified Hamacher product (*H*_1_, *H*_2_) for each of the groups is as follows:

(13) H1=μPxPγP∗μNxNγNμPxPγP+μNxNγN−μPxPγP∗μNxNγN.

*μ*_*P*_ is membership value of the platelet count, *γ*_*P*_ is concentration value for platelets, *μ*_*N*_ is membership value of the NS1 Panbio, and *γ*_*N*_ is dilution value for NS1 Panbio.

(14) $$\begin{array}{c} H_{2}=\frac{{\mu_{I}\left(x_{I}\right)}^{\gamma_{I}}*{\mu_{L}\left(x_{L}\right)}^{\gamma_{L}}}{\left({\mu_{I}\left(x_{I}\right)}^{\gamma_{I}}+{\mu_{L}\left(x_{L}\right)}^{\gamma_{L}}\right)-{\mu_{I}\left(x_{I}\right)}^{\gamma_{I}}*{\mu_{L}\left(x_{L}\right)}^{\gamma_{L}}}. \end{array}$$

*μ*_*I*_ is membership value of the IgG Panbio units, *γ*_*I*_ is concentration value for IgG Panbio, *μ*_*L*_ is membership value of the lymphocyte count, and *γ*_*L*_ is dilution value for lymphocyte count.

Concentration and the dilution of the membership values are carried out according to the relative importance that each of these parameters plays as an early indicator of severity. Platelet count and the IgG Panbio level play critical roles in the identification of the severity; thus their effects are concentrated. But NS1 Panbio level and lymphocyte counts are not early predictors of severity. Hence their effects are diluted. The values are assigned in an intuitive manner according to the information gained through medical expertise.

Accordingly the values assigned to *γ*_*P*_, *γ*_*N*_, *γ*_*I*_, and *γ*_*L*_ are 1.2, 0.2, 1.1, and 0.2, respectively.

The Hamacher operator value resulting from platelet count and the NS1 Panbio levels (13) and the Hamacher operator value gained from lymphocyte count and IgG Panbio units (14) are combined together using the OWA operator (2). Therefore the modified Ordered Weighted Aggregation operator is given by

(15) $$\begin{array}{c} OWA=\rho *\max\left\{\;H_{1},H_{2}\;\right\}+\left(\;1-\rho \;\right)*\min\left\{\;H_{1},H_{2}\;\right\}. \end{array}$$

For the OWA operator that is used in this research, only two weights are required and these two weights are constructed by assigning the value *a* = 0.1, *b* = 0.6 to the linguistic quantifier (4) and then applying to the weights formula (5). Hence *ρ* = 0.8.

Accordingly the orness (6) of the OWA operator that we have constructed is 0.8. Thus it can be seen that this operator is close to an OR operator which enables us to average the values given by the modified Hamacher operators for each parameter group.

#### 2.3.3. Determining the Ambiguous Region

The ambiguous region is determined using the ambiguous parameter levels which are mentioned earlier. The method we use to identify the ambiguous operator value range is as follows.

**Step 1 .**

The range of the ambiguous parameter values of each individual parameter is applied to the modified Hamacher operators of the relevant groups.

**Step 2 .**

The range of the values given by the modified Hamacher operators is calculated.

**Step 3 .**

The range of values given by the modified Hamacher product is applied to the OWA operator.

**Step 4 .**

The range of the values given by the OWA operator is taken as the ambiguous region of the operator.

**Figure 4:**
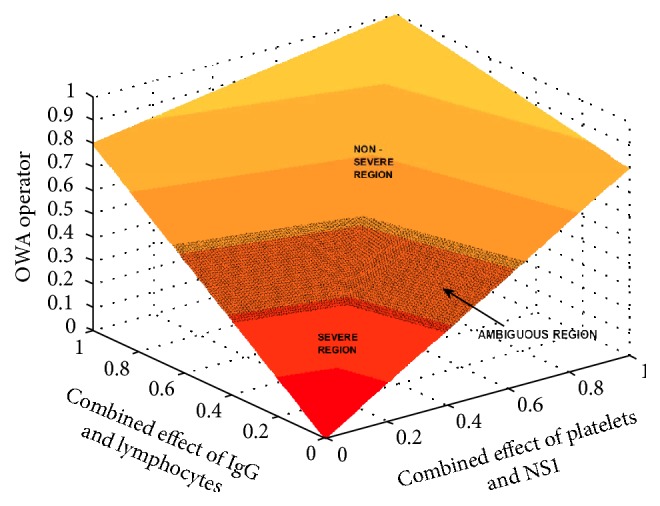
The operator with the ambiguous region.

**Figure 5:**
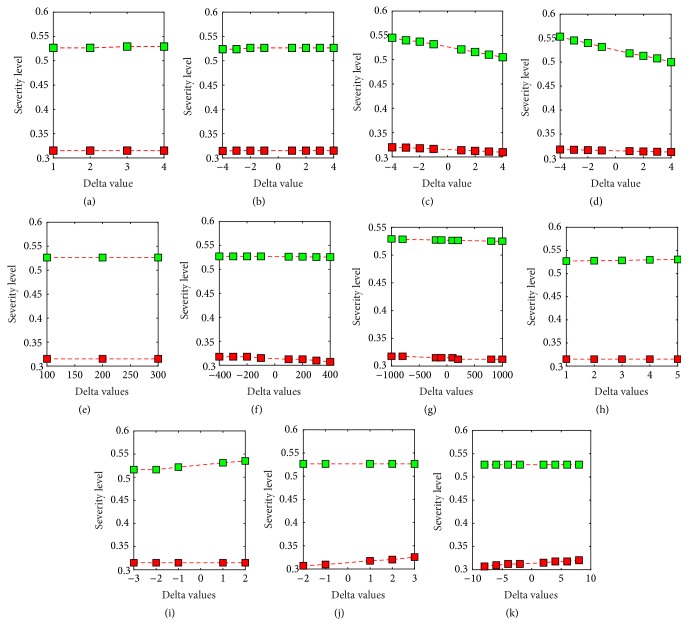
Change in the boundary values for a shift in the NS1 Panbio membership function at the NS1 value of 0 (a), NS1 Panbio membership function at the NS1 value of 75 (b), platelet count membership function at the platelet value of 25 (c), platelet count membership function at the platelet value of 150 (d), lymphocyte count membership function at the lymphocyte count of 100 (e), lymphocyte count membership function at the lymphocyte count of 1500 (f), change in the boundary values for a shift in the lymphocyte count membership function at the lymphocyte count of 6000 (g), IgG Panbio membership function at IgG value of 0 (h), IgG Panbio membership function at IgG value of 18 (i), IgG Panbio membership function at IgG value of 22 (j), and IgG Panbio membership function at IgG value of 100 (k).

The resulting partitions of the operator are illustrated in the Figure 4. Accordingly the ambiguous region lies between the values 0.3151 and 0.5264. The values above 0.5264 indicate the region with less severity whereas the region with higher severity is shown for values less than 0.3151.

## 3. Results and Discussions

### 3.1. Model Validation

The values from the data set are applied to the model and Table 3 indicates how accurately the model is able to classify the patients to severe and nonsevere categories. Table 3 summarizes the percentage of patients classified severe or nonsevere at the given time point.

In order to evaluate the performance of the model further we have to determine how many severe patients and nonsevere patients are classified correctly. Accuracy of classifying, classification as ambiguous, and misclassification are defined as follows:

(16) Accuracy  of  classifying=Severe  patients  classified  severe+Non  severe  patients  classified  non  severeTotal  number  of  patients

(17) Classification  as  ambiguous=DHF  patients  classified  ambiguous+DF  patients  classified  ambiguousTotal  number  of  patients

(18) Misclassification=DHF  patients  classified  non  severe+DF  patients  classified  severeTotal  number  of  patients

Accuracy of classifying is 52.78%. Through (16) we can observe that 36.11% of the patients are classified ambiguous. Equation (17) determines that 11.11% of the patients are misclassified. The patients who are classified ambiguous cannot be considered as a misclassification as the ambiguous region is an indication for the medical staff that the patient is heading into the severe region. The misclassification of 11.11% occurs due to the DF patients being classified as severe. The model is able to avoid a classification of severe patients as nonsevere, which would have been a more significant error. In general, it is an accuracy range from 53% to 89%.

### 3.2. Sensitivity Analysis

The objective of carrying out a sensitivity analysis is to identify whether classification of the patients according to the severity changes significantly in case of a shift in the degree of fuzziness.

**Figure 6:**
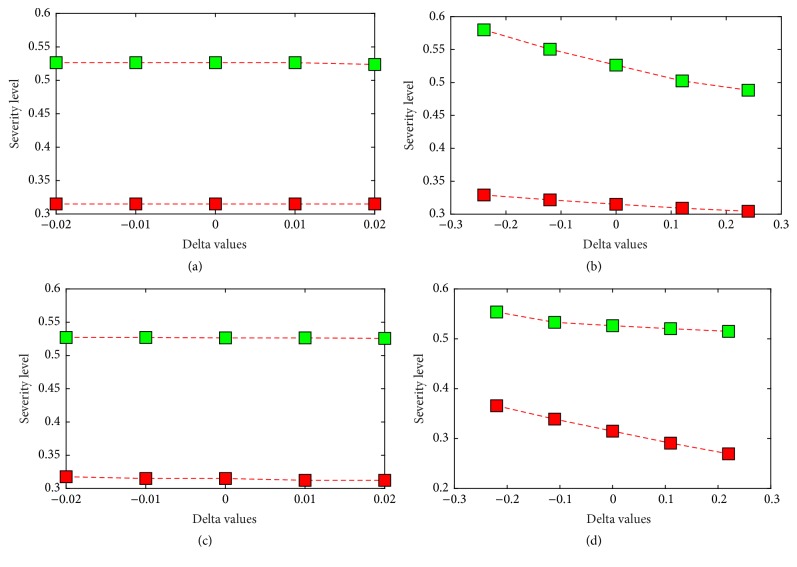
Change in the boundary values for a shift in the concentration of the NS1 membership values (a), change in the boundary values for a shift in the concentration of the platelet count membership values (b), change in the boundary values for a shift in the concentration of the lymphocyte count membership values (c), and change in the boundary values for a shift in the concentration of the IgG Panbio membership values (d).

Each of the membership functions has been shifted by a certain *δ* > 0 value and we observe the change in the boundary values for severe and nonsevere regions. The degree of change in the boundary value determines the robustness of the model and the relevant results are indicated in Figure 6 where the lower and upper boundary values are plotted according to the different *δ* values. According to Figure 6 it can be clearly seen that there is no significant change in the boundary values due to shift in any of the membership functions.

The concentrations and dilutions that are applied to each of the parameters are also shifted in order to identify the sensitivity of the model. The relevant results are illustrated through Figure 6. Figure 6 proves that a change in the concentrations and dilutions of the parameter membership values does not significantly alter the boundary values.

Since the boundary values do not change significantly for either a shift in the membership functions or concentrations it can be inferred that the accuracy levels of the model also remain relatively constant. Hence the model is not affected by changes in the degree of fuzziness which leads to the conclusion that the model is indeed robust.

## 4. Conclusions

Dengue is currently a significant issue not only in Sri Lanka but also in the South Asian region. Although the mortality rate of this disease is low, it can be further controlled by the developing a model which can predict the severity level of the patients.

Most of the research that is carried out so far focuses on predicting severity based on the information derived by a single parameter. In this research, the cumulative effects of the platelet count, NS1 Panbio levels, IgG Panbio levels, and lymphocyte count of the patient are considered. The statistical analysis shows that the behaviour of the platelet count and NS1 antigen levels are similar; furthermore the behaviour of the IgG antibody levels and lymphocyte count also indicates a similarity. The fuzzy logic principles involving fuzzy set theory and operator theory are used in order to construct the mathematical model which can predict the severity of the patient.

The model can be further improved by the inclusion of more parameters into the model. Cytokines and liver enzyme levels might be vital in increasing the accuracy of the model.

## Figures and Tables

**Table 1:** Descriptive statistics of the parameters.

Time point	DHF			DF		
	Mean	Median	Standard deviation	Mean	Median	Standard deviation
	Dengue specific IgG (Panbio units)					
96 h	39.51	30.3	39.06	13.03	9.3	15.09
108 h	48.1	37.2	45.3	20.9	10.1	28.7
120 h	45.9	40.8	45.8	37.8	37.6	34.8
132 h	50.4	34.8	51.5	47.2	46.4	41.7

	Lymphocyte count (cells/mm^3^)					
96 h	1012	764	696	814	710	257
108 h	1251	847	1095	1036.8	1011.6	139.8
120 h	1878	1499	1828	955	738	530
132 h	1719	1057	1401	2057	1463	1671

	Platelet count (cells/mm^3^)					
96 h	83.3	85.5	44.2	103.8	106	24
108 h	63.6	67	37.07	92.8	95.5	23.2
120 h	51	45.5	37.91	98.88	97	23.09
132 h	58.4	58.5	35.84	84.3	90	30.5

	NS1 antigen levels					
96 h	28.32	29.41	23.21	37.1	36.3	39.7
108 h	26.11	30.95	24.74	35.7	35.3	38.5
120 h	26.3	24.09	24.33	24.6	6.4	29.1
132 h	28.86	29.73	28.25	21.43	2.3	28.15

**Table 2:** Ambiguous parameter levels.

Parameter	Ambiguous region	
	Lower limit	Upper limit
Platelet count	70,000	95,000
NS1 antigen level	25	40
Lymphocyte count	2500	4000
IgG antibody level	16	36

**Table 3:** Accuracy of classifying DHF patients at different time points.

DHF					DF				
	Time point					Time point			
	96 hours	108 hours	120 hours	132 hours		96 hours	108 hours	120 hours	132 hours
Percentage of DHF patients classified severe	43.75%	53.33%	55%	42.11%	Percentage of DF patients classified severe	0%	0%	0%	28.57%
Percentage of DHF patients classified ambiguous	12.50%	26.67%	30%	57.89%	Percentage of DF patients classified ambiguous	66.67%	75%	28.57%	28.57%
Percentage of DHF patients classified nonsevere	43.75%	20%	15%	0%	Percentage of DF patients classified nonsevere	33.33%	25%	71.43%	42.86%
